# The *Trypanosoma cruzi *nucleic acid binding protein Tc38 presents changes in the intramitochondrial distribution during the cell cycle

**DOI:** 10.1186/1471-2180-9-34

**Published:** 2009-02-11

**Authors:** María A Duhagon, Lucía Pastro, José R Sotelo-Silveira, Leticia Pérez-Díaz, Dante Maugeri, Sheila C Nardelli, Sergio Schenkman, Noreen Williams, Bruno Dallagiovanna, Beatriz Garat

**Affiliations:** 1 Laboratorio de Interacciones Moleculares, Facultad de Ciencias, Montevideo, Uruguay; 2 Departamento de Genética, Facultad de Medicina, Montevideo, Uruguay; 3 Departamento de Biología Celular y Molecular, Facultad de Ciencias, Montevideo, Uruguay; 4 Departamento de Neurobiología Celular y Molecular, Instituto de Investigaciones Biológicas Clemente Estable Montevideo Uruguay; 5 Instituto de Investigaciones Biotecnologicas/INTECH, Universidad Nacional de General San Martin/CONICET, Buenos Aires, Argentina; 6 Departmento de Microbiologia, Imunologia e Parasitologia, Universidade Federal de São Paulo, São Paulo, Brazil; 7 Department of Microbiology and Immunology, University at Buffalo, Buffalo, USA; 8 Instituto de Biologia Molecular do Parana, Curitiba, Brazil

## Abstract

**Background:**

Tc38 of *Trypanosoma cruzi *has been isolated as a single stranded DNA binding protein with high specificity for the poly [dT-dG] sequence. It is present only in Kinetoplastidae protozoa and its sequence lacks homology to known functional domains. Tc38 orthologues present in *Trypanosoma brucei *and *Leishmania *were proposed to participate in quite different cellular processes. To further understand the function of this protein in *Trypanosoma cruzi*, we examined its *in vitro *binding to biologically relevant [dT-dG] enriched sequences, its expression and subcellular localization during the cell cycle and through the parasite life stages.

**Results:**

By using specific antibodies, we found that Tc38 protein from epimastigote extracts participates in complexes with the poly [dT-dG] probe as well as with the universal minicircle sequence (UMS), a related repeated sequence found in maxicircle DNA, and the telomeric repeat. However, we found that Tc38 predominantly localizes into the mitochondrion. Though Tc38 is constitutively expressed through non-replicating and replicating life stages of *T. cruzi*, its subcellular localization in the unique parasite mitochondrion changes according to the cell cycle stage. In epimastigotes, Tc38 is found only in association with kDNA in G1 phase. From the S to G2 phase the protein localizes in two defined and connected spots flanking the kDNA. These spots disappear in late G2 turning into a diffuse dotted signal which extends beyond the kinetoplast. This later pattern is more evident in mitosis and cytokinesis. Finally, late in cytokinesis Tc38 reacquires its association with the kinetoplast. In non-replicating parasite stages such as trypomastigotes, the protein is found only surrounding the entire kinetoplast structure.

**Conclusions:**

The dynamics of Tc38 subcellular localization observed during the cell cycle and life stages support a major role for Tc38 related to kDNA replication and maintenance.

## Background

*Trypanosoma cruzi *the protozoan responsible for Chagas disease belongs to a group of organisms that branched very early in eukaryotic evolution. Probably as a consequence of its evolutionary distance from higher eukaryotes, this protozoan shows several unique features. Among them, the mitochondrial DNA forms a unique network structure known as kinetoplast that is composed of two types of topologically catenated circular DNA molecules: maxicircles (20 to 37 kb) and minicircles (0.5 to 2.8 kb). The few dozens of maxicircles bear information equivalent to that of the mtDNA from higher eukaryotes while the several thousand diverse minicircles carry information for RNA editing in the form of guide RNA (gRNAs) that direct extensive modification of the maxicircle mRNA transcripts [1]. The replication of the kDNA is a complex process that takes place in a highly organized spatial and temporal pattern. It involves several kDNA replication specific proteins that have been mainly characterized in *T. brucei*, *Leishmania *and *Crithidia fasciculata *[2]. Several proteins associated with *T. cruzi *kDNA have also been reported (*i.e. *Hsp70 [3], KAP1 [4], Topoisomerase II [5], CRK1 [6], kDNABPs [7], UMSBP [8] and Calreticulin [9]). Recently, a 38 kDa protein (p38) of *T. brucei *[10] was proposed to participate in kDNA replication and maintenance. However, a different role was previously assigned for this protein. In fact, this protein (then named TbRBP38) and the *Leishmania tarentolae *orthologue (LtRBP38) were proposed as mitochondrial RNA binding proteins involved in non-specific modulation of mitochondrial RNA stability [11]. Concomitantly, we reported the isolation of the *T. cruzi *orthologue (Tc38) from nuclear enriched fractions [12]. We demonstrated that this protein has single stranded DNA binding abilities and that it shows a preferential binding to poly [dT-dG] sequences. In addition, the *Leishmania amazonensis *orthologous protein (LaGT2) was later purified from nuclear and S100 extracts using single stranded G telomeric oligonucleotide affinity chromatography [13]. Later it was suggested that the potential LaGT2 targets may not be restricted to telomere sequences [14].

[dT-dG] dinucleotides are well represented in nuclear DNA and also in mini and maxicircles. The minicircle replication origins include the universal minicircle sequence (UMS, GGGGTTGGTGTA) that is present in varying copy numbers and well conserved among different kinetoplastids [15,16]. The exact sequence of the maxicircle replication origin is not yet known although it has been mapped to the variable region of *T. brucei *and *C. fasciculata *[17]. Two copies of the UMS are present in the *T. brucei *variable region though they are absent in *T. cruzi *and *C. fasciculata *[18,19]. Interestingly, the *T. cruzi *maxicircle sequence [19] contains [dT-dG] rich tracts. The promoter sequence of *L. donovani *rDNA, which has also been involved in replication, is unusually rich in [dT-dG] repeats and bears an UMS homologue [20]. Replication origins are regions with the propensity to melt in order to facilitate the landing of the replication machinery while single stranded DNA binding proteins assist in the maintenance of the unwound state. In addition, single-stranded conformations mediated or maintained by [dT-dG] tracts in the nuclear DNA, and single stranded DNA binding proteins specifically recognizing them, could also favor the initiation and/or elongation of transcription. Indeed, single stranded DNA-protein interaction has been reported to affect the transcription of protein coding genes by RNA polymerase I [21]. The close association between elements that sustain transcription and replication is well documented [22]. Therefore potential nuclear/mitochondrial transcriptional/replication roles for Tc38 are likely.

To further understand the role of Tc38, we analyzed its binding specificity, expression levels and subcellular localization along life and cell cycle of *T. cruzi*. Our results indicate that although Tc38 is able to *in vitro *bind to several nuclear and mitochondrial [dT-dG] single strand sequences, it is essentially a mitochondrial protein. In addition, subcellular localization during the cell cycle is compatible with a major role for Tc38 in kDNA replication and maintenance.

## Results

### Native Tc38 is able to bind poly [dT-dG] and other [dT-dG] enriched targets

**Figure 1:**
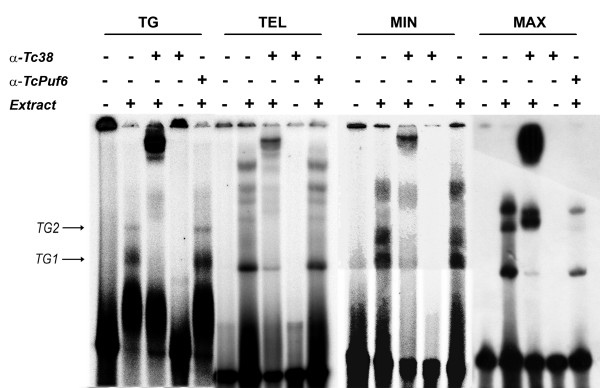
**Binding of native Tc38 to different [dT-dG] rich targets**. Whole protein extracts of exponentially grown epimastigotes cultures were assayed with oligonucleotide probes representing four putative targets: TG, TEL, MIN and MAX as indicated in Materials and Methods. Reactions were done under the conditions described in Materials and Methods using 1 μg of total epimastigote protein extract, 1 ng (10,000 cpm) of each probe. For each probe, we determined the presence of Tc38 in the complexes by the addition of the anti-Tc38 affinity purified polyclonal antibody (12 μL) to the binding reactions (α-Tc38). A polyclonal antibody against TcPuf6 (12 μL) was used as a control (α-TcPuf6). The presence/absence of the antibodies and protein extract in the binding reactions is indicated by +/- signs above each lane.

Using EMSA we previously identified two specific complexes (*TG*1 and *TG*2) arising from the interaction of epimastigote nuclear extracts with a [dT-dG]_40 _oligonucleotide probe [23]. Later we also showed that the recombinant purified Tc38-GST fusion protein was able to bind the same oligonucleotide probe [12]. To directly address the participation of the endogenous Tc38 in the initially reported nuclear extract complexes we performed EMSA supershift reactions. We employed a purified polyclonal antiserum raised against the recombinant GST-Tc38 protein that specifically recognizes a main band with an apparent molecular weight of about 38 kDa in total protein extracts of epimastigotes (see below). This antibody was able to supershift the complexes formed by the recombinant GST-Tc38 protein and the poly [dT-dG] probe (data not shown). As seen in Figure 1, complexes *TG1 *and *TG2 *were readily supershifted by this antibody. No supershift could be observed using the complementary oligonucleotide [dC-dA]_40 _as a probe (data not shown). These data indicate that Tc38 is present in the native protein complexes formed between the poly [dT-dG] probe and parasite extracts characterized previously [23] and favors its role in the *in vivo *sequence recognition.

Given the proposed roles in telomere and kinetoplast DNA recognition of Tc38 trypanosomatid orthologues, we analyzed whether endogenous Tc38 could also interact with single stranded [dT-dG] rich *cis*-acting sequences from nuclear and mitochondrial origins. Oligonucleotides containing the sequence of the telomere repeat, a [dT-dG] rich region of the *T. cruzi *maxicircle that is synthenically located to the replication origin mapped in *T. brucei *and the minicircle UMS were assayed *in vitro *by EMSA with whole *T. cruzi *epimastigote protein extracts. We observed a pattern of bands similar to that observed for the poly [dT-dG] probe (Figure 1) and these complexes were all supershifted by the anti-Tc38 antibody. Control reactions using the anti-TcPuf6 antibody [24] at the same concentration were unable to produce any supershift. These data suggest that native Tc38 is able to recognize single stranded [dT-dG] enriched sequences in different contexts and support a possible telomeric or kinetoplast-associated role.

### Tc38 is expressed throughout T. cruzi life cycle

**Figure 2:**
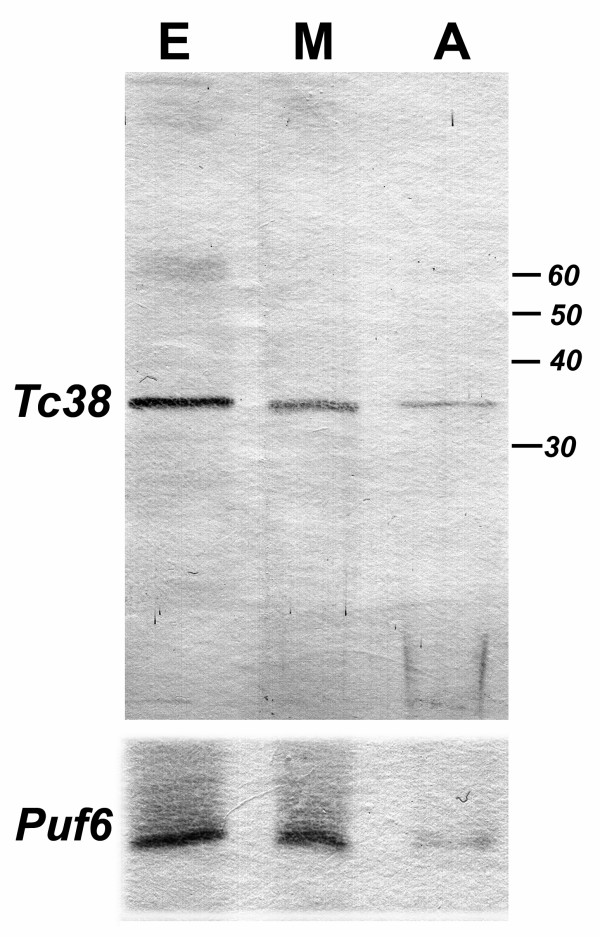
**Expression of Tc38 during the *T. cruzi *life cycle**. Western analysis of total protein extract using purified anti-Tc38 and anti-TcPuf6 antibody is shown. Protein extracts from 1 × 10^7 ^parasites were loaded into each lane. Life cycle stages are indicated as: E: epimastigotes, M: metacyclic trypomastigotes and A: amastigotes.

In order to better understand the Tc38 physiological role, we looked at its expression in both proliferative (epimastigotes and amastigotes) and non-proliferative (metacyclic trypomastigotes) stages of the parasite. The polyclonal antiserum raised against GST-Tc38 was used to probe membranes with total protein extracts from different stages by western analysis. As shown in figure 2, a band of 38 kDa was observed in all extracts from the various parasite life cycle stages. Normalization of Tc38 levels was performed using TcPuf6, another RNA binding protein, which showed minimal variation during *T. cruzi *life cycle [24].

### Tc38 is found in the T. cruzi mitochondrion

Tc38 bears a hypothetical N-terminal mitochondrial targeting signal and its orthologous genes in *T. brucei *and *L. tarentolae *have been proposed to encode mitochondrial proteins [11]. TbRBP38/p38 has also been shown to co-localize with the kinetoplast in a *T. brucei *transfectant overexpressing the fusion protein p38-GFP [10]. However, other researchers have isolated orthologues from a *L. amazonensis *nuclear enriched fraction and/or for its affinity for nuclear DNA targets [13]. These data together with Tc38 ability to bind kinetoplastid and telomeric sequences could be integrated by proposing a dual localization of this protein, both in the mitochondrion and the nucleus.

**Figure 3:**
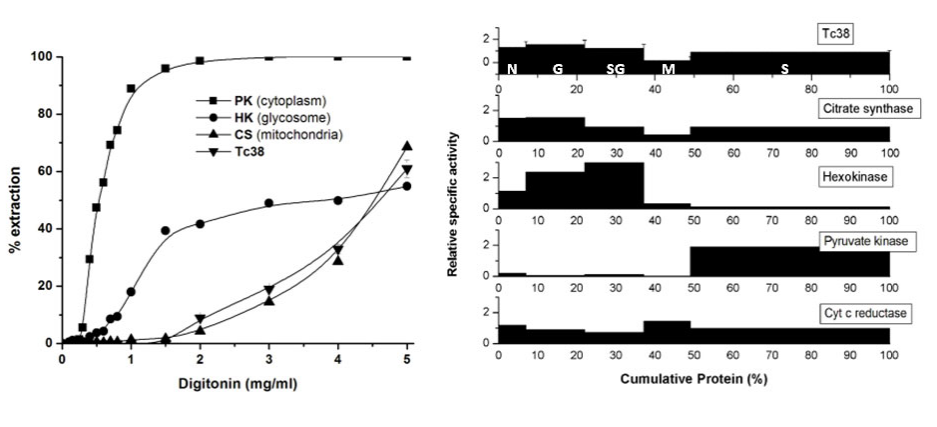
**Subcellular localization of Tc38 using biochemical approaches in *T. cruzi *epimastigotes**. (A) Digitonin extraction. Epimastigotes (125 mg per tube) were incubated with different digitonin concentrations (indicated on the abscissa) as described in Materials and Methods. Marker enzymes activities: hexokinase (HK), citrate synthase (CS), and pyruvate kinase (PK). The amounts of Tc38 were determined by western analysis. (B) Subcellular fractionation. The experiment was carried out using 3.3 g (wet weight) of parasites. Fractions are plotted in the order of their isolation, from left to right: nuclear (N), large granule (G), small granule (SG), microsomal (M) and supernatant (S). The ordinate represents relative specific activity (percentage of total activity/percentage of total protein). The abscissa indicates the cumulative protein content. The percentage of recovery for the marker enzymes: citrate synthase 70.9%, hexokinase 74.1%, cytochrome C reductase 43.6%, pyruvate kinase 85.3%, Tc38 61.1%. Error bars indicate the variation in band intensity seen by quantification of the western blot.

In order to address Tc38 localization we pursued two biochemical approaches. Firstly, we performed a stepwise digitonin extraction of intact epimastigote cells. The pattern of Tc38 extraction was compared with those of cytosolic (PK), mitochondrial (CS), and glycosomal (HK) markers (Figure 3A). The Tc38 extraction curve clearly follows that of CS. It begins to be extracted at a digitonin concentration of 2.0 mg/mL, and at 5 mg/mL 39% of the protein still remained in the pellet. This pattern supports the hypothesis of a predominant mitochondrial localization of Tc38 in the cell.

Secondly, we carried out subcellular fractionation experiments. They also showed that Tc38 is a mitochondrial protein since the highest specific activity was observed in the large granular fraction (Figure 3B). The recovery of CS activity in the nuclear fraction suggests a contamination of this fraction with mitochondrial proteins.

### Tc38 presents a complex pattern of distribution within the mitochondrion

**Figure 4:**
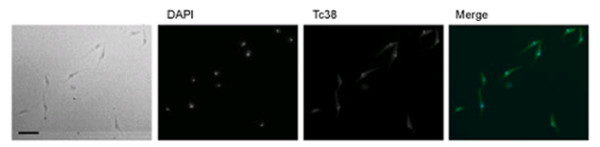
**Subcellular localization of Tc38 using immunohistochemical approaches in asynchronous cultures of *T. cruzi *epimastigotes**. DAPI staining and Tc38 signal are indicated. Left panel shows the pooled ISIS software (MetaSystems GmbH) captured image. For the merge image, Tc38-Alexa 488 signal is shown in green and DAPI nucleic acid staining in blue. Bars = 10 μm.

In order to address the subcellular localization of Tc38 with another approach we performed immunohistochemistry. The analysis of asynchronous epimastigote cultures showed a non-homogeneous Tc38 pattern (Figure 4). Parasites exhibit a widespread dotted distribution in an area that resembles the branched shape of the mitochondrion. In addition, 75.8 ± 0.5% (n = 500) cells present a strong Tc38 staining on the kinetoplast. As commonly seen in epimastigotes, DAPI brightly stains the "disk" shaped kinetoplast DNA and produces a weak signal in the rounded nuclear DNA. Although control experiments using nuclear protein antibodies verified the penetration of the antibodies into the nucleus (data not shown), we were unable to detect any consistent nuclear fluorescence from Tc38 in these preparations.

### Tc38 intramitochondrial distribution changes during the cell cycle

Since Tc38 was found to predominantly co-localize with the kDNA and to recognize single stranded mini and maxicircle replication related sequences, we focused on the intramitochondrial localization during the cell cycle.

**Figure 5:**
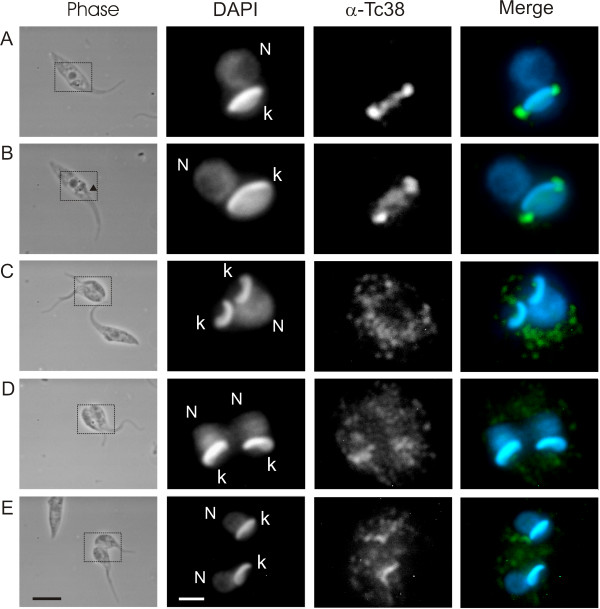
**Tc38 patterns in *T. cruzi *epimastigotes during the cell cycle**. Phase contrast, DAPI staining and Tc38 signal are indicated. For the merge images, Tc38-Alexa 488 signal is shown in green and DAPI nucleic acid staining in blue. Selected parasites that show the most frequent patterns seen in the cell cycle phases are presented. A corresponds to G1/S, B to G2 and C to E show images from mitosis to cytokinesis. Each one of the Tc38 labeling patterns were found in the majority of examined cells (n ≥ 20). The arrow indicates the position of the second flagellum, indicative of G2. Black bars = 5 μm. The dotted lines in the phase contrast indicate the position enlarged in the fluorescent images. White bars = 2 μm.

For this purpose, we first analyzed asynchronic cultures. We based the identification of each cell cycle stage on morphological markers including both the number of nuclei and kinetoplasts determined by DAPI staining together with the number and appearance of flagella assessed by phase contrast microscopy [25]. Figure 5 shows the sequential changes in Tc38 localization during the cell cycle. It shows that G1/S cells usually exhibit a homogeneous signal over the kDNA (Figure 5A) even though in some cases Tc38 condenses in two small antipodal sites. Cells at G2 (see arrow showing the second flagellum in phase contrast image) exhibit a diffuse signal connecting what now has become two clearly defined spots (Figure 5B). The two Tc38 spot signals do not seem to exactly co-localize with the DAPI staining. As the cell cycle progresses the defined spots of Tc38 disappear and the diffuse dotted signal spreads out, covering a region far beyond the kinetoplast and without an evident association with it (Figure 5C and 5D). Finally in late cytokinesis the signal of Tc38 tends to regain the homogenous distribution over the kDNA (Figure 5E).

**Figure 6:**
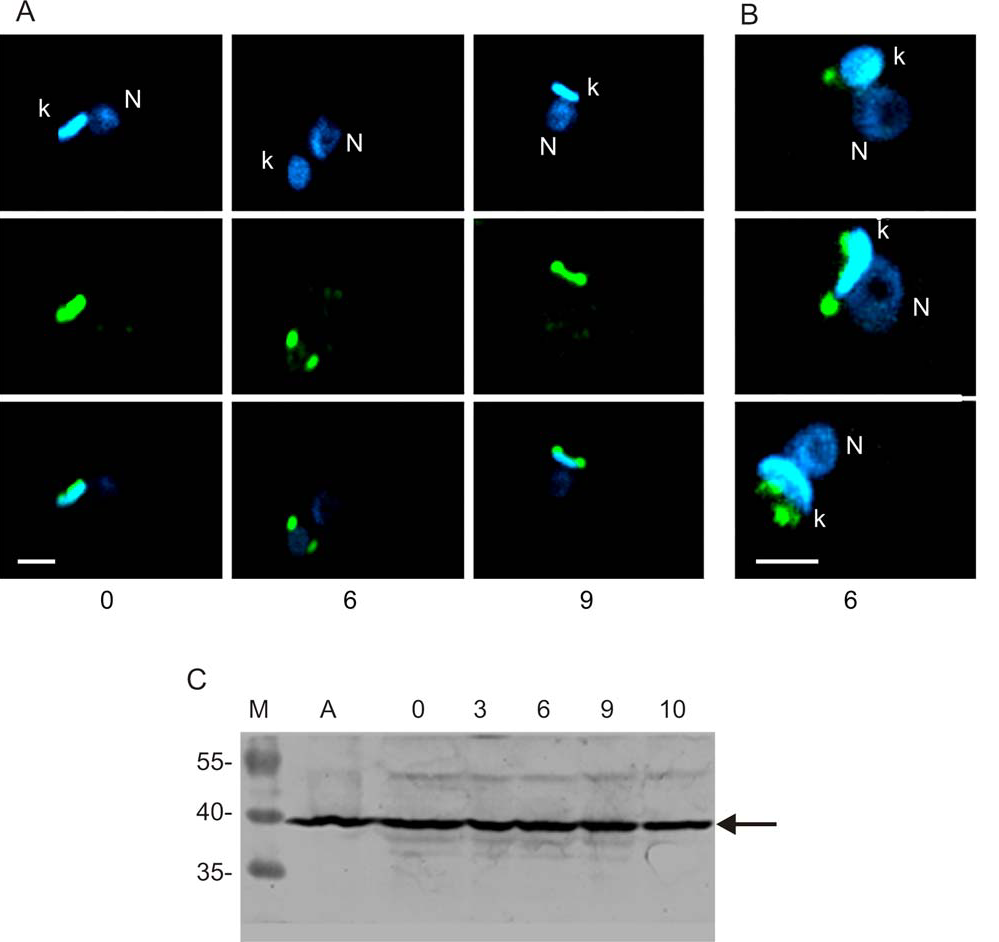
**Tc38 patterns in *T. cruzi *epimastigotes synchronized with hydroxyurea**. Tc38-Alexa 488 signal is shown in green and DAPI nucleic acid staining in blue. "N" indicates the nucleus and "k" indicates the kinetoplast. (A) Single confocal sections (~0.3 μm thick) of selected parasites that show the most frequent patterns seen in the cell cycle progression after hydroxyurea removal, at the indicated times. Upper panels show the DAPI blue signal, middle panels the Tc38 signal and bottom panels the merge of both. The same patterns were observed in three different synchronization experiments. (B) Z projection of 31 optical sections (~0.3 μm thick) of three selected parasites at 6 h after HU removal. Only the merge of the DAPI and Alexa-488 signals is shown. (C) Western blot of total protein extract using purified anti-Tc38 antibody. M: molecular weight markers, A: protein extracts of asynchronous epimastigote cultures in exponential growth phase. Remaining lanes correspond to protein extracts of epimastigote cultures after removal of HU at the times (hours) indicated above each lane. 1 × 10^7 ^cells were loaded onto each lane. Molecular weights of the protein ladder are indicated on the left of the gel (kDa).

We also studied Tc38 localization in cultures synchronized with hydroxyurea (HU). HU inhibits the enzyme ribonucleotide reductase and the resulting depletion of deoxyribonucleotides arrests DNA replication in late G1/early S phase [26]. Previous reports on the effects of HU treatment on the *T. cruzi *cell cycle phases considered S phase to occur between 3–6 h and G2 at 9 h after HU removal [27,28]. Progression of the cell cycle was followed using the same time schedule. We obtained images that are in agreement with the Tc38 sub-cellular localization determined in asynchrony (Figure 6). At time 0 we found that cells generally show a homogeneous signal over the kDNA (Figure 6A). Among them, a small percentage of the cells present two intense signals generally associated with the kinetoplast DNA. At 3–6 h, cultures largely present two defined spots flanking the kDNA disk and the images at 10 h also exhibit a signal connecting them. Further quantitative analyses are required to determine the significance of each distinct pattern contribution. Interestingly, as indicated above, the Tc38 staining at 6 h after HU removal does not co-localize with the DAPI staining, being mainly adjacent to the kDNA disk. In fact, higher resolution confocal images of cultures indicate that Tc38 localizes near but not on the kDNA (Figure 6B). Images of either non-synchronized or HU synchronized cells show quite similar patterns in more than 200 parasites.

Tc38 content during the epimastigote cell cycle was also studied by western analysis using protein extracts from HU treatment (Figure 6C). Even though a constant major band corresponding to Tc38 molecular weight is observed, additional faint bands are also detected.

### Tc38 presents a dynamic distribution during the parasite life cycle

**Figure 7:**
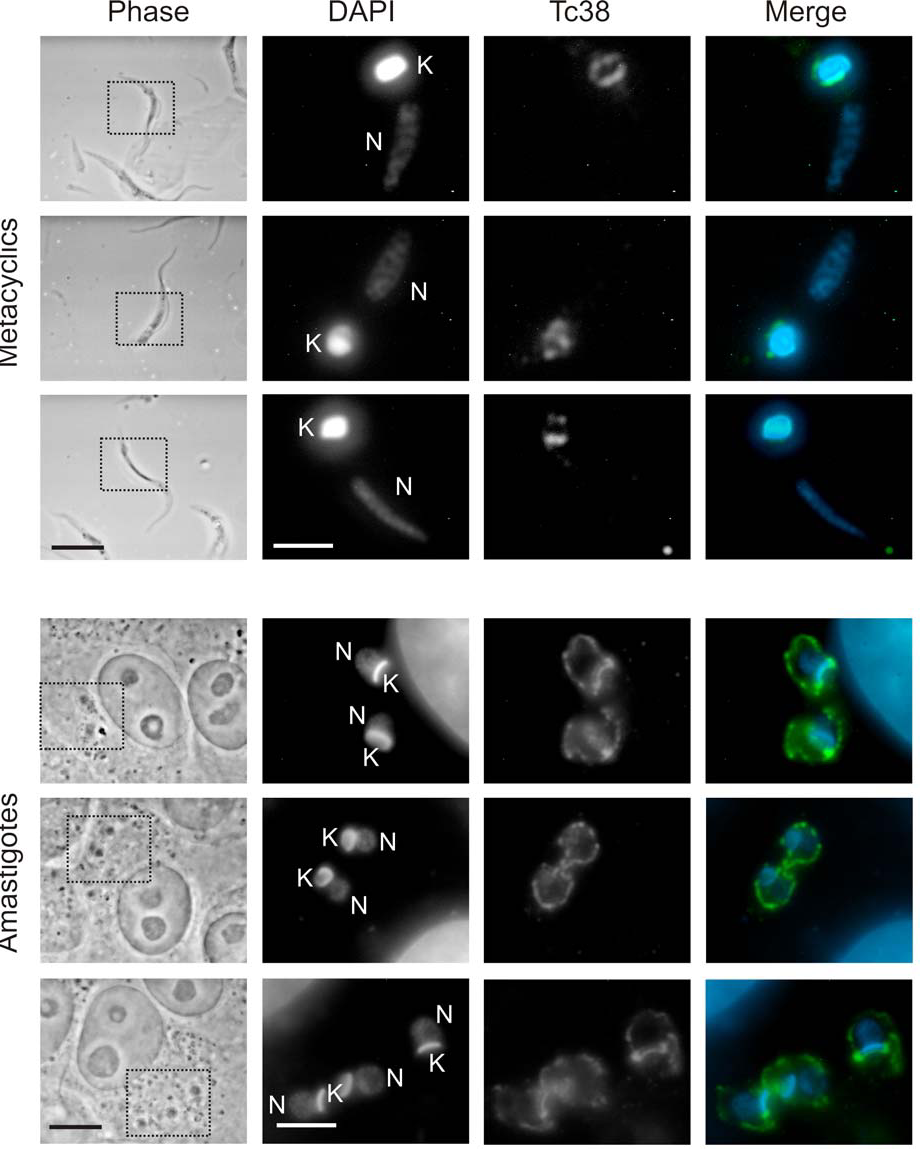
**Tc38 patterns in *T. cruzi *metacyclic trypomastigotes and amastigotes**. Phase contrast, DAPI staining and Tc38 signal are indicated. For the merge images, Tc38-Alexa 488 signal is shown in green and DAPI nucleic acid staining in blue. "N" indicates the nucleus and "K" indicates the kinetoplast. Selected metacyclic trypomastigotes and amastigotes that show the most frequent patterns observed are presented. Bars = 5 μm. The dotted box in the phase contrast corresponds to the position of the fluorescent images.

To further understand the putative role of Tc38, we compared the labeling pattern of replicative epimastigotes with proliferative amastigotes and non-proliferative metacyclic trypomastigotes (Figure 7). In the non-replicative metacyclic form, Tc38 is always found surrounding the kinetoplast. However, in the replicative amastigote, Tc38 showed a dynamic localization; whereas some amastigotes exhibit a widespread distribution that is not restricted to the kDNA (as seen in epimastigotes during the late G2, M and C phases), others present a homogenous Tc38 signal associated with the kDNA. Taken together these results suggest that Tc38 changes the internal localization pattern only in the replicative stages of *T. cruzi *life cycle.

## Discussion

We had previously reported the isolation of Tc38 as a novel single stranded DNA binding protein without known functional domains [12]. It bears a well-defined N-terminal mitochondrial targeting signal and the orthologous protein in *T. brucei *has been proposed to be a mitochondrial RNA binding protein [11] and more recently to associate with the kDNA [10]. Here we found that anti-Tc38 causes a specific supershift of the complexes formed by total protein extracts of *T. cruzi *and [dT-dG] rich oligonucleotides including [dT-dG]_40_, the Universal Minicircle Sequence, a repeated maxicircle sequence putatively related to replication, and the telomere repeat.

Biochemical data obtained with both digitonin titration and differential centrifugation suggested that Tc38 preponderantly resides in the mitochondrion. The fact that Tc38 presents an extraction profile similar to citrate synthase indicates that it is a soluble matrix protein. Therefore, the previous isolation of Tc38 from nuclear enriched fractions in *T. cruzi *[12] and its orthologous protein in *L. amazonensis *[13], and the identification of a 38 kDa putative minicircle DNA binding protein in *T. cruzi *nuclear extracts [7], could be explained by the contamination of the nuclear fraction with fragmented mitochondria. In fact, there seems to be an intimate association between the mitochondrial and the nuclear membrane in the proximity of the kinetoplast in epimastigotes. The extent of mitochondrial contamination could be masking a putative nuclear localization if the protein nuclear abundance is low.

The subcellular localization of Tc38 shown by immunohistochemistry was consistent with the biochemical data, and further evidenced the association with the kinetoplast, depending on the cell cycle stage. The analysis of Tc38 distribution in asynchronic cultures and in parasites obtained with the *T. cruzi *culture synchronization elegantly described by Galanti et al. [27] indicates that Tc38 localization within the mitochondrion is not static. Yet, exit from the mitochondria in mitosis cannot be excluded. Tc38 shows a homogeneous distribution in G1, a discrete antipodal position in S and a more extended location including the antipodes and the kDNA between them in G2. The antipodal localization of Tc38 disappears in late G2 turning to a diffuse dotted signal extending beyond the kinetoplast. It is worth mentioning that although many replication protein change their abundance along the cell cycle, some others, such as the universal minicircle sequence binding protein (UMSBP) and DNA polymerase β are constitutive [29]. Studies of the timing of nuclear and mitochondrial DNA synthesis and segregation [25,29] had shown that nuclear S phase correlates with kDNA S phase (kS), G2 corresponds to the end of replication and the beginning of the segregation of the already replicated kDNA, M nuclear phase has already separated kinetoplasts and G1 correlates to the early kS. We interpret the Tc38 homogeneous signal as corresponding to the kinetoplast G1 phase. In addition, the dumbbell pattern might correspond to kDNA replication itself. When the segregation of the kDNA is complete, Tc38 signals exhibit a dotted and extended location that is maintained during the subsequent replication and segregation of the nuclear DNA. Approaching the kinetoplast G1 phase, Tc38 reorganizes over the kDNA. Indeed the proportion of positive cells exhibiting the Tc38 staining over the kDNA could represent cells in nuclear G1, S and early G2 phases accounting for approx. 76% of the cell cycle.

The punctate distribution over the mitochondrial matrix in cells approaching mitosis and during cytokinesis could also account for a particular distinctive role of the protein. Alternatively it could be a result of inefficient kDNA targeting and/or association. Interestingly, the presence of DNA derived from kDNA (aDNA) in the matrix has been previously reported [30]. In addition, a similar pattern has been described for proteins involved in kDNA replication and maintenance [31]. Given the ability of Tc38 to also bind RNA, it would be interesting to investigate whether the foci correspond to RNPs engaged in the transport or translation of mitochondrial RNAs. To our knowledge there is no report on the RNA and RNPs redistribution in the mitochondria of trypanosomatids.

The subcellular localization of Tc38, its ability to bind mini and maxicircles sequences related to replication, the implication of the *T. brucei *orthologous protein in the kDNA replication, and our results showing a dynamic localization of Tc38 implicate the protein in cell cycle progression. Current models of kDNA replication propose that minicircles stretched parallel to the axis of the disk shaped kinetoplast are released from the network and initiate replication at the kinetoplast flagellar zone [1]. The progeny then migrate to the antipodal sites where they are reattached to the network. In *T. cruzi *they attach uniformly to the periphery (annular) in contrast to the antipodal (polar) reattachment observed in *T. brucei *and *C. fasciculata *[32]. In this context it is possible that Tc38 is initially recruited at both replication enzymatic complexes at kS phase and later it becomes bound to newly replicated minicircles that are positioned in an annular fashion around the kDNA disk. By the end of replication Tc38 might be located on the two segregating kinetoplasts. This distribution could account for a different non-replicative role of the protein in structural or dynamic processes of the kDNA structure. We do not clearly understand the sequence of the transition from the homogeneous G1 to the antipodal and more elongated distribution of the protein in S/G2.

Given the ability of Tc38 to bind to [dT-dG] rich repeats contained in maxicircle replication regions, a possible involvement in the replication process cannot be ruled out. It is worth mentioning that overgrown epimastigote cultures show groups of parasites that completely lack the Tc38 signal on the kDNA. This could mean that Tc38 is not at the kDNA in a G0-like stage triggered by environmental conditions. Indeed, we cannot exclude the possibility that Tc38 could be released from the kDNA at a physiological G1, later being recruited when the cell enters the S phase.

The constant levels of the 38 kDa protein detected by western analysis of HU synchronized cultures suggest that it does not undergo major covalent modifications that could explain the Tc38 dynamics. These data might suggest a passive role of the protein in the movement around the kDNA disk, being guided by other proteins that actively participate in the motor process and/or the cycle timing control. Otherwise a subtle modification of a minor pool of protein itself would be responsible for changes in its localization. Perhaps, the additional bands on the western blot seen in the HU treated parasites could represent covalent modifications of the protein engaged in the replicative process of the kDNA.

Finally, our immunochemical assays did not detect Tc38 in the nucleus in different phases of the cell cycle. We still cannot completely rule out a discrete nuclear distribution tightly restricted to a phase not visible after the hydroxyurea synchronization or too short to be significantly represented in the cultures. However, the failure to see a clear nuclear signal in the asynchronic cultures does not support the hypothesis of a dual localization. In addition, the absence of conspicuous covalent modifications of the protein that could account for different subcellular localization or intra-compartmental distribution reinforces this interpretation. Unless higher resolution studies should prove the contrary, the data here presented strongly support the hypothesis of an exclusively mitochondrial localization.

## Conclusion

The *Trypanosoma cruzi *nucleic acid binding protein Tc38 is able to bind single stranded [dT-dG] enriched sequences from nuclear and mithocondrial DNA. Nevertheless, different approaches established that it predominantly localizes to the unique parasite mitochondrion. Although Tc38 is constitutively expressed, it shows a dynamic localization in the proliferative parasite forms that could implicate the protein in events dependent on the cell cycle. Taken together our data strongly suggest that Tc38 is an exclusively mitochondrial protein which has a role in the kinetoplast biology, perhaps in the replication or segregation processes.

## Methods

### Parasite culture

Unless specified, the *T. cruzi *Dm28 clone was used for the experiments. Epimastigotes were cultured to exponential growth phase in liver infusion tryptose (LIT) liquid medium [33] supplemented with 10% heat inactivated fetal calf serum (Sigma), 0.025 mg/mL hemin, 30 μg/mL streptomycin and 50 μg/mL penicillin at 28°C. Metacyclic trypomastigotes were obtained according to Contreras *et al*. [34]. Briefly, epimastigotes in late exponential growth phase were harvested by centrifugation and incubated for two hours at 28°C in artificial triatomine urine medium (TAU; 190 mM NaCl, 17 mM KCl, 2 mM CaCl_2_, 2 mM MgCl_2_, 8 mM phosphate buffer pH 6.0) at a density of 5 × 10^8 ^cells/mL. Thereafter, the parasites were incubated in TAU3AAG medium (TAU supplemented with 10 mM L-proline, 50 mM L-glutamate, 2 mM L-aspartate, 10 mM glucose) to a final concentration of 5 × 10^6 ^cells/mL. After incubation at 28°C for 72 h, the parasites were resuspended in PSG (73 mM NaCl, 1% glucose, 5 mM sodium phosphate, pH 8.0) and separated in DEAE-52-cellulose [35]. The metacyclic trypomastigotes obtained were recovered by centrifugation and resuspended in TAU medium. They were then treated for 30 min at 37°C with an equal volume of fresh guinea pig serum. After washing the parasites 3 times with NKM buffer (40 mM NaCl, 5 mM KCl, 2 mM MgCl_2_, 10 mM HEPES, pH 7.4), they were used to infect VERO cells in a 10:1 parasite: VERO cell ratio. The infected monolayers were cultured in RPMI medium (SIGMA) at 37°C without agitation in a 5% CO_2 _atmosphere for 4 days. After 24 h of infection the medium was changed daily. Four-day-old infected monolayers of VERO cells containing amastigotes were transferred to a 37°C incubator without CO_2 _supply. After approximately two days, disrupted cells released the intracellular amastigotes. They were purified from the cell debris by allowing them to decant in sterile 50 mL Falcon tubes and/or by centrifugation at 1,000 × g for 5 min. The calculated purity of the different developmental stages was between 90–100%. Protein extracts were prepared as previously described [36].

### Tc38 Antibody

A polyclonal antiserum (anti-Tc38) was raised in New Zealand White rabbits by immunization with the recombinant fusion protein GST-Tc38 using Freund's adjuvant. Rabbits were inoculated sub-cutaneously three times, at two-week intervals, with the protein (250 μg each time) and serum was obtained two weeks after the last boost. The polyclonal serum was purified on DEAE Affi-Gel^®^Blue columns (BioRad) following manufacturer's instructions. Afterwards, purification using protein extract of *T. cruzi *epimastigotes and *E. coli *protein extract bound to Affi-Gel 10 Gel columns (BioRad) was performed. 1 mL of Affigel-10 was washed with H_2_0 and incubated with 24 mg (8 mL) of whole *T. cruzi *(A) or *E. coli *(B) protein extract dialyzed against 0.1 M MOPS pH 7.5, for 2 h with gentle rocking. Next, 0.1 mL of 1 M glycine ethyl ester pH 8 was added to reaction, incubated for 1 h at 4°C and thoroughly washed with 1 × PBS. Then, 4.5 mL of the DEAE Affi-Gel^®^Blue purified serum (2 mg/mL) was added to the resin and incubated for 1.5 hours at room temperature with gentle rocking. The resin was decanted by gravity and the supernatant of column B was recovered. This antibody fraction was used in western blot assays. For column A, the supernatant was discarded and the antibody fraction bound to the *T. cruzi *extract was eluted by the addition of 1 mL of 0.1 M glycine-HCl pH 2.3 after previous washes in PBS-Tween 1% (10 mL three times) and one wash with PBS. The eluate was collected in 0.2 mL of 1 M Tris-HCl pH 11 for a quick neutralization and was stored at 4°C with 0.2% sodium azide. This antibody fraction was used in EMSA experiments. The anti-TcPuf 6 antibody used in the experiments was the serum fraction purified by DEAE Affi-Gel^®^Blue [24].

### Western blot

Protein extracts were separated by electrophoresis in 12.5% SDS-polyacrylamide gels and electro-transferred onto ECL membranes (GE Healthcare) following standard procedures. Membranes were blocked by incubation in 5% skim milk powder in buffer PBS-0.1% Tween and were then incubated for 1 h at room temperature with the polyclonal antibody purified by procedure B (described above) diluted 1:500. Bound antibodies were detected using peroxidase conjugated AffiniPure goat anti-rabbit IgG (H+L) (Jackson Immuno Research) diluted 1/2,500, with the color reaction developed using 5 mg of DAB (Sigma) in 10 mL 0.05 M Tris pH 7.6 and 10 μL 30% H_2_O_2_.

### Binding reactions

Total protein extract from *T. cruzi *epimastigotes was obtained by centrifuging and washing, exponentially growing cultures, in PBS at 1,000 × g for 10 min at 4°C. After three washes in 1 volume of PBS the pellet was resuspended in lysis buffer (10 mM Tris-HCl pH 7.5, 1 mM EDTA, 1 mM EGTA, 5 mM DTT, 10% glycerol and protease inhibitors) to a final density of 1 × 10^8 ^cells/mL. After 5 pestle strokes at 2,000 rpm in a Tri-R Stir-R homogenizer (Model K41), 0.75% CHAPS was added to the buffer and the mix was incubated for 30 min on ice with gentle rocking. The solution was finally centrifuged at 4°C, 23,000 × g for 30 min in order to remove cell debris. Total protein concentration was determined using the Protein Assay reagent (BioRad). The electrophoretic mobility shift assay (EMSA) was done essentially as previously reported [23]. Binding reactions were incubated at room temperature for 20 min in 20 μL reaction volume containing: binding buffer (10 mM Tris-HCl, 10 mM KCl, 10 mM MgCl_2_, 1 mM DTT, 1 mM EDTA), 5 mM spermidine and 0.2 μg of poly [dI-dC] [dI-dC] as a non-specific competitor, and immediately loaded onto a 6% native polyacrylamide gel. Incubation of the extracts with antibodies and unlabelled oligonucleotides were carried out for 10 min prior to the addition of the labeled probe. The reaction was left at room temperature for 20 more min. The sequences of the four oligonucleotides used were: TGTGTGTGTGTGTGTGTGTGTGTGTGTGTGTGTGTGTGTG (TG_20_), GGGTTAGGGTTAGGGTTAGGGTTAGGGTTAGGGTTA (TEL), GGGGTTGGTGTAGGGGTTGGTGTAGGGGTTGGTGTA (MIN) and TTAAATAGTAGTGTTGTTTAACCTTAAATAGTAGTGTTGTTTAACC (MAX). The probes were end labeled with [γ^32^P]dATP using T4 polynucleotide kinase and purified by MicroSpin G-50 columns (Amersham Bioscience). 1 ng of oligonucleotide was used for each binding reaction.

### Digitonin treatment

A preliminary assessment of the subcellular localization of the enzymes was made by digitonin treatment of intact parasite cells as reported [37]. Briefly, epimastigotes of the *T. cruzi *CL Brener clone were suspended in 25 mM Tris-HCl buffer pH 7.6, containing 1 mM EDTA and 0.25 M sucrose, 10 μM E-64 with the addition of a freshly prepared digitonin solution at final concentrations of up to 3 mg/mL. After incubation at 25°C for 5 min, the cells were separated by centrifugation and the supernatants were kept for enzyme assays. The pellets were suspended in the same buffer and sonicated. Enzymatic activities of marker enzymes for mitochondria, glycosomes, and cytosol were determined in both fractions. 100% activity was taken as the sum of the activities in both fractions at a given digitonin concentration. The protein concentration of Tc38 was determined by western analysis following the procedure described above. The relative quantification of Tc38 in western blots was performed using a standard curve composed of serial dilutions of a *T. cruzi *protein extract in the linear range of intensity. The membranes were scanned at 600 dpi and the band intensities were calculated using the software IDScan EX v3 1.0 (Scanalytics, Inc.) as the Gaussian integrated density. The presented values are the average intensity of three serial dilutions of each fraction in the linear range of intensity from three technical replicate experiments.

### Cell fractionation by centrifugation

The subcellular localization was also studied by differential centrifugation [37]. The fractions obtained were: nuclear fraction (N, 1,000 × g, 10 min), large granules (LG, 7,600 × g, 10 min), small granules (SG, 27,000 × g, 20 min), microsomal fraction (M, 200,000 × g, 1 h) and the soluble fraction (C). The latter contains the cytosol as well as soluble proteins leaking out of damaged organelles. The pellets were washed three times and suspended in 1.1 mL of the same buffer used for the digitonin experiments. The activities of marker enzymes for mitochondria, glycosomes, microsomes and cytosol, and protein concentration of Tc38, were determined as described above.

### Biochemical markers for subcellular compartments

The enzymatic activities were assayed at 30°C; the reaction mixtures were equilibrated for 3 min at this temperature, and the reactions were usually started by addition of the cell-free extract. Citrate synthase (CS, mitochondrial marker) [38], hexokinase (HK, glycosomal marker), NADPH cytochrome C reductase (CR, microsomal marker) and pyruvate kinase (PK, cytosolic marker) [39] were assayed as previously described [37].

### Immunohistochemistry

For immunohistochemistry, parasites were harvested from culture media, washed four times and resuspended with PBS (2 × 10^6 ^cells/mL) and deposited on poly-lysine coated slides. They were fixed with 2% paraformaldehyde in PBS for 15 min at 4°C, permeabilized by three short incubations in PBS-0.1% Triton-X100 followed by blocking with PBS-0.1% Triton-X100-1% BSA for 30 min. The slides were then incubated with the primary antibody (anti-Tc38) in PBS-0.1% Triton-X100-0.1% BSA, washed three times and then incubated with the secondary antibody anti-rabbit Alexa-488 F(ab') fragment of goat anti-rabbit IgG (H+L) (Molecular Probes). Incubations were done overnight at 4°C or alternatively for 4 h at 37°C. Total DNA staining was achieved using DAPI (10 μg/mL) for 10 min at room temperature. Slides were then mounted in 1 part of Tris-HCl pH 8.8 and 8 parts of glycerol. Confocal images were acquired at room temperature using a Zeiss LSM 510 NLO Meta system (Thornwood, NY, USA) mounted on a Zeiss Axiovert 200 M microscope using either an oil immersion Plan-Apochromat 63×/1.4 DIC objective lens or Plan-Apochromat 100×/1.4 DIC. Excitation wavelengths of 488 nm and 740 nm (2-photon laser from Coherent) were used for detection of the green signal and DAPI, respectively. Fluorescent emissions were collected in a BP 500–550 nm IR blocked filter and a BP 435–485 nm IR blocked filter, respectively. All confocal images were of frame size 512 × 512 pixels or 1024 × 1024, scan zoom range of 1–5.5 and line averaged 4 times.

### Cell synchronization

Synchronization of cells was essentially done as described [27]. In brief, cells were grown to a density of 0.5 – 1 × 10^7 ^cells/mL, washed twice in 1 volume of PBS at 4°C (700 × g without brake) and incubated for 24 h at 28°C in LIT medium containing 20 mM hydroxyurea (HU). Cells were then identically washed, resuspended in fresh LIT medium without HU and incubated at 28°C for different time intervals. Finally, they were washed three times in PBS at 4°C and fixed for immunohistochemistry. Based on prior reports on the effects of HU treatment on the *T. cruzi *cell cycle phases [27,28] we considered S phase to occur between 3–6 h after HU removal.

## Authors' contributions

MAD designed the experiments, set up the techniques, performed the experimental approaches, data analysis and interpretation and writing of the manuscript. LPD and BD performed the first purification of the Tc38 antibody and collaborated in the assessment of Tc38 expression through life cycle by western blot. Digitonin solubilization and subcellular fractionation was performed by MAD and DM at Dr. J.J. Cazzulo's lab. LP assisted with the immunofluorescence assays. JRSS did the confocal analysis and provide helpful guidance to set up Tc38 immunohistochemistry protocol. Immunofluorescence analysis of Tc38 in the life cycle stages as well as during cell cycle in asynchronic cultures of epimastigotes was done by LP and SN at SS's lab. SS, BD and NW participated in helpful discussions of the results and critical reading of the manuscript. NW also collaborated in outlining the experimental strategies. BG conceived and designed the project, mentored MAD and reviewed the manuscript for its publication. All authors read and approved the final manuscript.
